# Editorial: Cognitive Diagnostic Assessment for Learning

**DOI:** 10.3389/fpsyg.2021.806636

**Published:** 2021-11-30

**Authors:** Peida Zhan, Feiming Li, Hong Jiao

**Affiliations:** ^1^College of Teacher Education, Zhejiang Normal University, Jinhua, China; ^2^Key Laboratory of Intelligent Education Technology and Application of Zhejiang, Zhejiang Normal University, Jinhua, China; ^3^Measurement, Statistics and Evaluation, Department of Human Development and Quantitative Methodology, University of Maryland, College Park, MD, United States

**Keywords:** cognitive diagnosis, longitudinal cognitive diagnostic assessment, assessment for learning, computerized adaptive test (CAT), cognitive diagnosis model (CDM)

Measuring and improving individual development are actively tackled in psychological, educational, and behavioral sciences. In the past decades, cognitive diagnosis (Leighton and Gierl, 2007), which objectively quantifies students' current learning status and provides diagnostic feedback, has been increasingly needed in different settings to measure and improve individual development.

Although cognitive diagnosis aims to promote student learning based on diagnostic feedback and the corresponding remedial intervention, currently, only a few studies have focused on and evaluated the effectiveness of such feedback or remedial intervention (e.g., Wang et al., 2020; Tang and Zhan, 2021; Wang S. et al.). One of the main reasons is that most cognitive diagnoses adopt a cross-sectional design. This issue may also be reflected in the cognitive diagnosis models (CDMs) or diagnostic classification models (for review, see von Davier and Lee, 2019), the primary tools for data analysis in cognitive diagnosis. Although various CDMs have been proposed, they are only applicable to cross-sectional data analysis (see von Davier and Lee, 2019).

By contrast, longitudinal cognitive diagnosis evaluates students' knowledge and skills and identifies their strengths and weaknesses over a period of time. The data collected from longitudinal learning for diagnosis allow researchers to develop models for learning tracking, which can be used to track individual growth over time and evaluate the effectiveness of feedback. Compared to cross-sectional learning diagnosis, longitudinal cognitive diagnosis may provide an additional perspective to evaluate student learning when aiming to promote student learning.

Currently, longitudinal cognitive diagnosis (e.g., Li et al., 2016; Zhan et al., 2019) mainly stays in the model development stage and lacks practical applications and related research on issues such as missing data, measurement invariance, and linking methods. Moreover, although some longitudinal CDMs have been proposed, these models still have limitations that need further exploration and improvement.

This Research Topic intends to highlight issues, practices, and methodologies dealing with evaluating and improving individual growth in learning, especially using cognitive diagnosis. This Research Topic presents the cutting-edge research related to quantitative methods and applications related to student development (e.g., the development of longitudinal CDMs, the development of longitudinal diagnostic assessments, learning progression, and the impact of sample attrition), novel CDMs for specific test situations (e.g., random guessing behavior, rater effects, and mixed format assessments), theoretical issues in cognitive diagnosis (e.g., parameter estimation, Q-matrix specification, and non-parametric classification method), and application issues in adaptive testings (e.g., automated test assembly, item exposure control, online calibration, and attribute coverage). The contributions of this special topic are elaborated as follows.

First, new quantitative methods and applications related to student development were proposed. Wen et al. proposed the HMM/ANN longitudinal CDM, in which the artificial neural network (ANN) was used as the measurement model of the hidden Markov model (HMM) to realize longitudinal tracking of students' cognitive skills. Pan et al. proposed a multivariate longitudinal CDM, in which the log-linear cognitive diagnostic model as the measurement model component evaluates the mastery status of attributes at each measurement occasion, and a generalized multivariate growth curve model that describes the growth of each attribute over time. Lin et al. proposed longitudinal CDMs that incorporate latent growth curve modeling and covariate extensions to measure the growth of skills mastery and evaluate attribute-level intervention effects over time. Tian et al. proposed a longitudinal CDM for hierarchical attributes by imposing model constraints on the transition CDM. In addition, Wang S. et al. reported developing and evaluating a learning program that integrated a longitudinal diagnostic assessment with two different learning interventions to diagnose and improve mental rotation skills. Furthermore, Bai and Wu et al. showed how to use CDMs to explore students' learning progression. Moreover, Pan and Zhan examined the impact of a common type of sample attrition, namely individual-level random attrition, on longitudinal cognitive diagnosis through a simulation study.

Second, novel CDMs for specific test situations were proposed. Choi et al. presented an approach in which the CDM was used with a statistical topic model to analyze item responses in mixed format assessments (i.e., multiple-choice and constructed-response items). Further, to estimate rater effects on constructed response times, Li X. et al. proposed CDMs within the frameworks of facets models and hierarchical rater models, using the log-linear cognitive diagnosis model as a template. Moreover, considering some students may engage in rapid guessing without thoughtful consideration on some items, Hsu et al. proposed a CDM with item response and response time to model rapid guessing behavior and enhance cognitive diagnosis.

Third, some theoretical details of cognitive diagnosis have also been concerned. Zhang et al. proposed a highly effective Pólya-Gamma Gibbs sampling algorithm to estimate the DINA model based on auxiliary variables. Furthermore, Wang W. et al. proposed a semi-supervised learning approach and an optimal design for examinee sampling for Q-matrix specification under the conjunctive and disjunctive model with an independent structure. In addition to parametric models, non-parametric diagnostic methods are also an essential method in cognitive diagnosis. Guo et al. introduced a non-parametric spectral clustering algorithm to cluster students according to their responses.

Fourth, although classification accuracy is critical in cognitive diagnostic computerized adaptive testing (CAT), attention has increasingly shifted to item exposure control to ensure test security and attribute balance/coverage to ensure test fairness. In such cases, Sun et al. developed the binary restrictive threshold method to balance measurement accuracy and item exposure. Wang Y. et al. proposed the attribute discrimination index-based method to balance the attribute coverage. Furthermore, online calibration is a technique to calibrate the parameters of new items in CAT, which seeds new items in answering operation items and estimates the parameters of new items through the response data of examinees on new items. Xiong et al. extended the two most popular calibration methods, one- and multiple EM cycle methods, to the graded response model for polytomous data. Moreover, Li G. et al. explored the automated test assembly in cognitive diagnostic multistage adaptive testing that can be seen as a combination of the paper and pencil-based test and CAT.

Finally, Zhan reviewed the current status and possible future research directions of longitudinal cognitive diagnosis. He pointed out that there are still many issues related to longitudinal cognitive diagnosis worthy of discussion. For example, (a) only binary attributes (e.g., “1” means mastery and “0” means non-mastery) were considered in most current studies. In the future, the polytomous attributes (Karelitz, 2004) or probabilistic attributes (Zhan et al., 2018) can be incorporated into longitudinal CDMs to track students' refined development (e.g., Zhan, 2021); (b) only item response accuracy data were considered in most current studies. In the future, utilizing multimodal data (e.g., item response times and eye-tracking indices) can evaluate the growth of students in multiple aspects, which is conducive to a more comprehensive understanding of the development of students (e.g., Wang et al., 2018); (c) most current studies assumed that attributes are structurally independent. However, when attribute hierarchy (Leighton et al., 2004) exists, the development trajectory of students is not arbitrary and should be developed in such hierarchical order. Therefore, incorporating the attribute hierarchy into current longitudinal CDMs is worth exploring (e.g., Zhan and He, 2021), and (d) adaptive learning and testing system involving longitudinal CDMs is also worthy of further study.

With 19 papers from 62 authors, this topic enhances interdisciplinary research fields such as psychometrics, pedagogy, psychology, statistics, computer science, educational technology, to name a few. The categorization focused on each paper's core contribution though some papers can be cross-classified. The papers' key findings and advancements well-represent the current state-of-the-art in the field of longitudinal cognitive diagnosis in educational and psychological assessments. As topic editors, we are happy to receive such a great collection of papers with various foci and make these publications right when the concept of assessment for/as learning is rapidly gaining popularity. We hope these papers fill some gaps in the literature related to longitudinal cognitive diagnosis modeling and applications. It is expected that the methodological papers will inspire more researchers to explore new frontiers in models and methods for longitudinal cognitive diagnosis; in the meantime, the methodological innovation will guide practitioners to improve their practices.

## Author Contributions

PZ contributed to manuscript drafting and revising. FL and HJ contributed to manuscript revising. All authors contributed to the article and approved the submitted version.

## Funding

This work was supported by the MOE (Ministry of Education in China) Project of Humanities and Social Sciences (Grant No. 19YJC190025) and the National Natural Science Foundation of China (Grant No. 31900795).

## Conflict of Interest

The authors declare that the research was conducted in the absence of any commercial or financial relationships that could be construed as a potential conflict of interest.

## Publisher's Note

All claims expressed in this article are solely those of the authors and do not necessarily represent those of their affiliated organizations, or those of the publisher, the editors and the reviewers. Any product that may be evaluated in this article, or claim that may be made by its manufacturer, is not guaranteed or endorsed by the publisher.
